# Transformer-based intelligent detection model for early dental caries in panoramic radiographs

**DOI:** 10.1038/s41598-025-33391-y

**Published:** 2026-01-23

**Authors:** Liwei Wang, Zhiyuan Li

**Affiliations:** Department of Stomatology, General Hospital of PLA Northern Theater Command, Shenyang, 110002 Liaoning China

**Keywords:** Dental caries detection, Transformer architecture, Panoramic radiography, Deep learning, Early diagnosis, Medical image analysis, Computational biology and bioinformatics, Diseases, Health care, Mathematics and computing

## Abstract

Early detection of dental caries in panoramic radiographs remains challenging due to subtle radiographic features and complex anatomical structures. This study develops a Transformer-based intelligent detection model specifically optimized for identifying early-stage carious lesions in panoramic dental images. The proposed architecture integrates enhanced multi-scale feature fusion mechanisms, spatially-aware attention optimization, and improved two-dimensional positional encoding to capture global contextual relationships while maintaining fine-grained feature discrimination. A comprehensive dataset comprising 3,856 panoramic radiographs with 12,847 annotated carious lesions across severity grades (D1-D4) was constructed for model development and validation. The model achieved 87.3% mean average precision (mAP) across all caries stages, with notable sensitivity of 81.3% for D1 lesions and 84.7% for D2 lesions, surpassing conventional CNN-based approaches and average dentist performance. The system processes images in real-time (70 milliseconds per radiograph). This research demonstrates the efficacy of domain-adapted Transformer architectures for early dental caries detection and establishes its potential utility as a decision support tool for enhancing diagnostic accuracy and screening efficiency in dental practice.

## Introduction

Dental caries remains one of the most prevalent chronic diseases worldwide, affecting approximately 2.3 billion people with untreated caries in permanent teeth^1^. Early detection and intervention of carious lesions are crucial for preventing disease progression and preserving tooth structure^2^. Panoramic radiography, as a routine diagnostic tool in dental practice, provides comprehensive visualization of the entire dentition and surrounding structures in a single image, making it an invaluable resource for caries screening^3^. However, the interpretation of panoramic radiographs heavily relies on the expertise and experience of dental practitioners, leading to variability in diagnostic accuracy and potential oversight of early-stage lesions^4^.

Recent advances in artificial intelligence, particularly deep learning algorithms, have demonstrated remarkable capabilities in medical image analysis^5^. Convolutional Neural Networks (CNNs) have been successfully applied to dental caries detection, achieving diagnostic performance comparable to experienced dentists^6^. Nevertheless, CNNs exhibit inherent limitations in capturing long-range dependencies and global contextual information due to their local receptive field characteristics^7^. The Transformer architecture, originally proposed for natural language processing, has emerged as a powerful alternative by leveraging self-attention mechanisms to model global relationships within data^8^. Its application in computer vision tasks has shown superior performance in capturing spatial dependencies and contextual features^9^.

While bitewing radiographs are considered the gold standard for detecting proximal caries due to their superior spatial resolution and minimal anatomical overlap, panoramic radiography offers distinct advantages that justify its selection as the primary imaging modality for this AI-assisted screening system. Panoramic radiographs provide comprehensive visualization of the entire dentition and surrounding structures in a single exposure, making them routinely used in initial dental examinations, particularly for population-based screening programs and patients with limited access to comprehensive dental care. Studies have demonstrated that panoramic radiography achieves comparable diagnostic accuracy to intraoral radiographs for posterior caries detection, particularly in molar regions, while offering superior patient acceptance and reduced procedural complexity. Furthermore, the development of AI systems specifically optimized for panoramic images addresses an underserved clinical need, as these radiographs are already widely integrated into standard dental practice workflows. The proposed system is designed to function as a preliminary screening tool that can identify areas requiring further investigation with more detailed imaging modalities such as bitewing radiographs, thereby optimizing clinical resources and enhancing overall screening efficiency.

Despite these technological advances, several critical challenges persist in automated caries detection systems. First, existing methods predominantly focus on bitewing or periapical radiographs, with limited exploration of panoramic images that present unique complexities including anatomical overlap and varying image quality^10^. Second, early-stage carious lesions exhibit subtle radiographic features that are difficult to distinguish from normal tooth structures, resulting in high false-negative rates^11^. Third, the scarcity of large-scale annotated datasets for panoramic dental images hinders the development and validation of robust detection models.

The necessity of developing an intelligent detection system specifically designed for early caries identification in panoramic radiographs is multifaceted. Such a system would enhance screening efficiency, improve detection consistency across varying levels of expertise, and enable early detection for timely preventive interventions.

This study develops a Transformer-based intelligent detection model for early dental caries in panoramic radiographs. The main research contributions include: (1) proposing a novel Transformer architecture optimized for panoramic dental image analysis with enhanced feature extraction capabilities for early carious lesions; (2) constructing a large-scale annotated dataset of panoramic radiographs with detailed caries annotations at various stages; (3) implementing multi-scale attention mechanisms to address the challenge of detecting subtle early-stage lesions; (4) conducting extensive evaluation to assess the model’s diagnostic performance and reliability^12^.

## Background

### Panoramic radiographic image characteristics and caries pathological analysis

Panoramic radiography captures the entire maxillofacial region in a single exposure but produces geometric distortions, magnification artifacts, and overlapping anatomical structures, resulting in reduced spatial resolution compared to intraoral radiographs^13–15^. Importantly, overlapping structures in the premolar region represent a well-recognized limitation that can obscure proximal caries detection. Early dental caries require 30–40% mineral loss before becoming radiographically detectable, appearing as subtle radiolucent areas^16,17^. Table 1 summarizes the standardized caries grading system.

**Table 1:** Caries classification standards and radiographic feature Comparison.

Grade	Clinical classification	Radiographic features	Detection complexity
D0	Sound tooth structure	No radiolucency, intact enamel density	Low
D1	Initial enamel caries	Subtle radiolucency in outer enamel, minimal contrast	Very High
D2	Established enamel caries	Visible radiolucent zone, confined to enamel	High
D3	Dentin caries	Radiolucency extending into dentin-enamel junction	Moderate
D4	Deep dentin caries	Extensive radiolucency approaching pulp chamber	Low

Manual radiographic interpretation is subjective and experience-dependent, with sensitivity for early caries detection in panoramic radiographs ranging from 0.37 to 0.69^18–20^. These limitations underscore the need for automated detection systems with high sensitivity and reproducibility.

### Deep learning applications in medical image detection

Convolutional Neural Networks have revolutionized dental radiographic analysis by automatically extracting hierarchical features, achieving accuracies exceeding 90% for tooth classification tasks^21–23^. Segmentation architectures such as FCN and U-Net have also been applied to dental imaging^27,28^. Object detection architectures including Faster R-CNN and YOLO have been successfully adapted for dental lesion localization^24–26^. However, CNNs exhibit inherent limitations: (1) most studies focus on bitewing rather than panoramic radiographs, (2) detection performance degrades for early-stage lesions, and (3) local receptive fields limit capacity for capturing long-range spatial dependencies essential for contextual understanding in complex anatomical regions^29,30^. These constraints motivate exploring Transformer architectures that model global contextual relationships while maintaining sensitivity for early caries detection.

### Transformer architecture principles and vision applications

The Transformer architecture uses self-attention mechanisms to model global dependencies^31^:

$$\:Attention\left(Q,K,V\right)=softmax\left(\frac{Q{K}^{T}}{\sqrt{{d}_{k}}}\right)V$$

where $\:Q$, $\:K$, and $\:V$ represent query, key, and value matrices, enabling each position to attend to all others simultaneously. Positional encoding compensates for permutation invariance^33,34^, while multi-head attention projects into multiple subspaces for diverse perspectives^35,36^:

$$\:MultiHead\left(Q,K,V\right)=Concat\left({head}_{1},...,{head}_{h}\right){W}^{O}$$

Vision Transformer (ViT) adapts this for images by partitioning into patches, achieving state-of-the-art results with superior efficiency^37,38^. Unlike CNNs’ local receptive fields, Transformers’ global attention captures long-range dependencies beneficial for detecting subtle features across large anatomical regions in panoramic radiographs^39^.

## Methods

### Overall model architecture design

The proposed model adopts a hierarchical Transformer architecture optimized for early caries detection, integrating multi-scale feature extraction with spatial attention mechanisms^40^. Preprocessing includes histogram equalization, bilateral filtering, and normalization. Images are resized to $\:512\times\:512$ pixels. Data augmentation (rotation $\:\pm\:15{\text{}}^{\circ\:}$, horizontal flipping, intensity variation) improves generalization^41,42^.

The feature extraction module employs a pyramid Transformer encoder^43^. Images are partitioned into $\:16\times\:16$ pixel patches, generating $\:32\times\:32$ tokens embedded into 768 dimensions. The encoder comprises 12 layers with 12 attention heads, extracting hierarchical features at layers 3, 6, 9, and 12 to capture multi-scale representations.

**Fig. 1:**
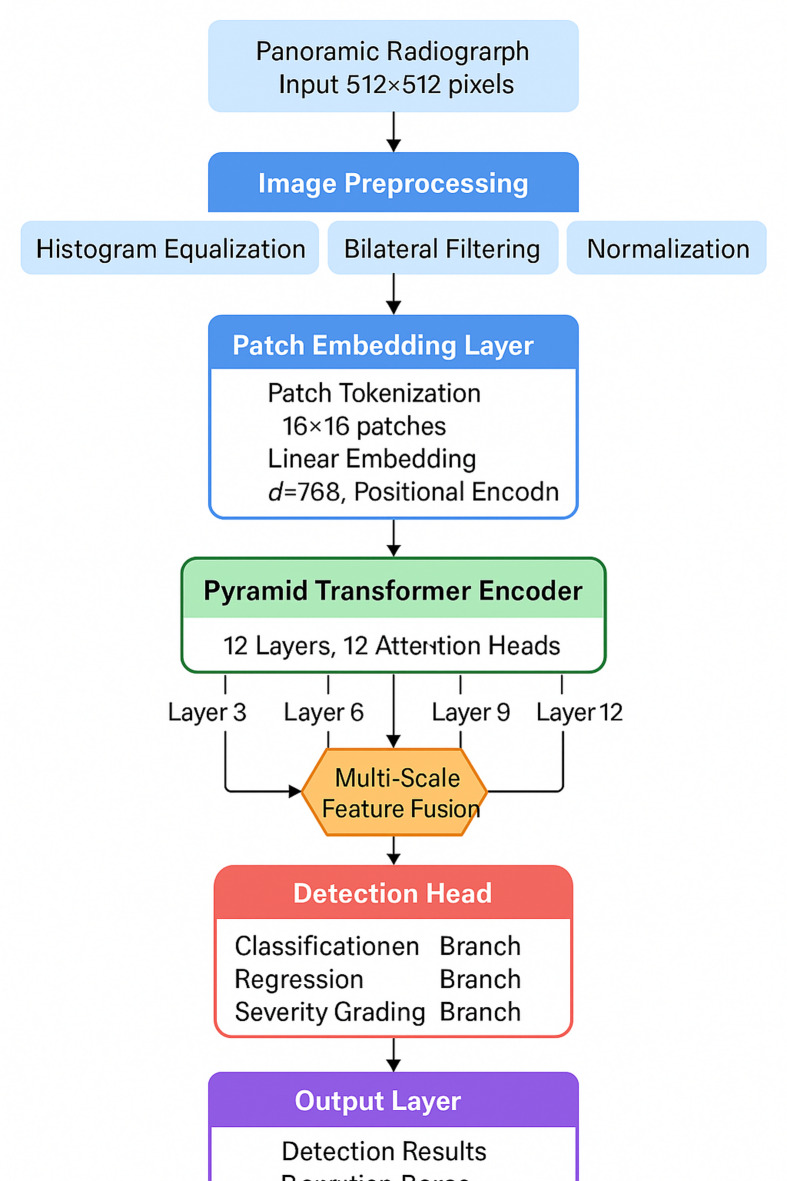
Overall architecture of the Transformer-based caries detection model.

Figure 1 shows the detection head utilizing Feature Pyramid Network structure^44^. The loss function combines three components:

$$\:{L}_{total}={\lambda\:}_{1}{L}_{cls}+{\lambda\:}_{2}{L}_{box}+{\lambda\:}_{3}{L}_{focal}$$

where $\:{L}_{cls}$ is classification loss, $\:{L}_{box}$ is bounding box regression loss, and $\:{L}_{focal}$ is focal loss addressing class imbalance^45^, with $\:{\lambda\:}_{1}=1.0$, $\:{\lambda\:}_{2}=2.0$, $\:{\lambda\:}_{3}=1.5$.

### Enhanced transformer encoder design

The encoder incorporates domain-specific modifications for panoramic dental radiographs^46^. Multi-scale feature fusion integrates features from different depths through weighted aggregation, with learnable weights dynamically computed to emphasize relevant features at each severity level^47^. Spatially-aware attention optimization enhances focus on tooth regions while suppressing irrelevant structures^48^. The 2D-aware positional encoding captures spatial relationships more effectively than standard 1D encoding^49^. Feature enhancement modules implement sequential channel-wise and spatial attention to refine features. Table 2 lists key configuration parameters.

**Table 2:** Model parameter Configuration.

Parameter	Value	Description
Input resolution	512 × 512	Standardized image size
Patch size	16 × 16	Patch dimension for tokenization
Embedding dimension	768	Feature vector dimension
Encoder layers	12	Number of Transformer blocks
Attention heads	12	Multi-head attention count
Dropout rate	0.1	Regularization probability
Learning rate	1e-4	Initial optimizer learning rate

**Fig. 2:**
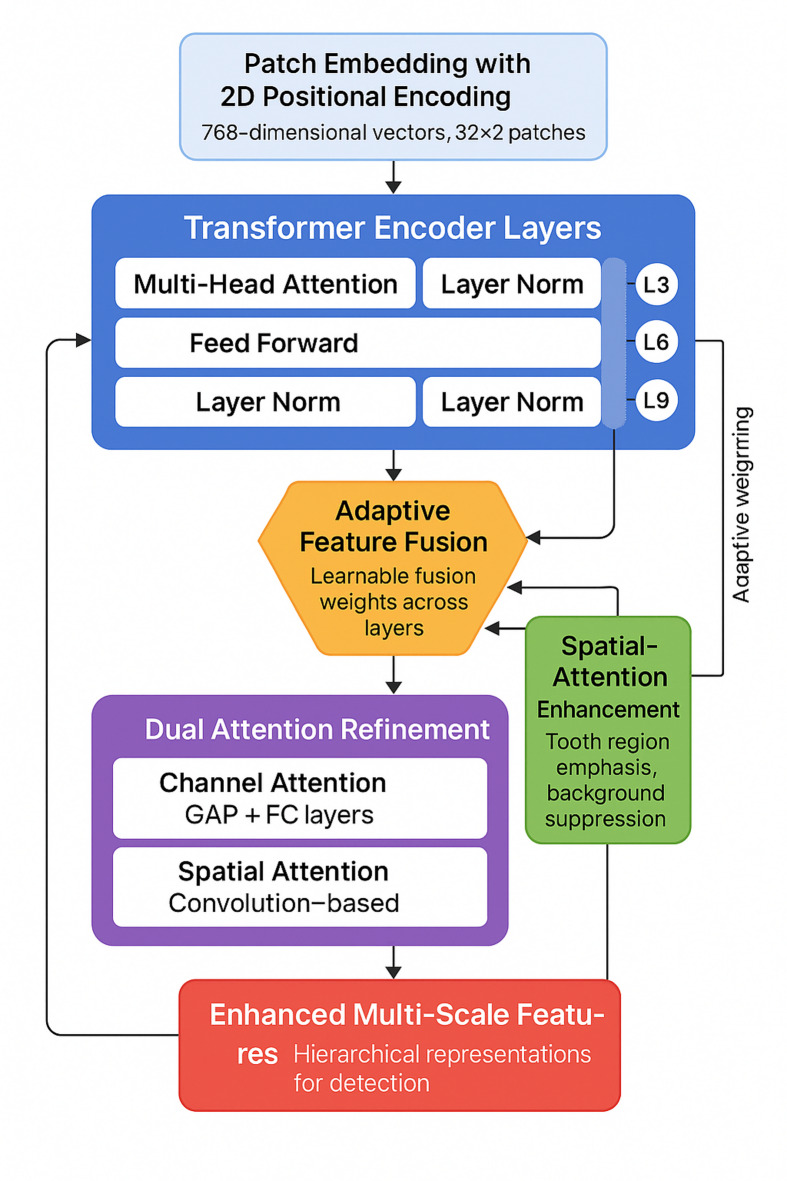
Enhanced transformer encoder architecture with multi-scale fusion and spatial attention mechanisms.

Figure 2 demonstrates the enhanced encoder structure, illustrating the hierarchical feature extraction pathway and the integration of multi-scale fusion modules at intermediate layers.

### Detection decoder and post-processing strategy

The decoder comprises three parallel branches for classification, bounding box regression, and severity grading^51^. Bounding box regression uses GIoU loss for improved gradient information^52^. Training follows a two-stage approach: ImageNet pretraining for 50 epochs, then end-to-end fine-tuning for 200 epochs using AdamW optimizer with cosine annealing schedule^53^. Post-processing employs soft-NMS with Gaussian decay to preserve valid detections for adjacent teeth. Adaptive confidence thresholds (0.3 for D1-D2, 0.5 for D3-D4) maximize sensitivity for early lesions while maintaining specificity. Mixed precision training enables batch size 16 on NVIDIA V100 GPUs^54^.

## Results

### Dataset construction and experimental setup

Panoramic radiographs were collected from three tertiary dental hospitals between January 2021 and December 2023, encompassing 3,856 patients aged 18–75 years^55^. All images were acquired using standardized digital panoramic X-ray systems (Planmeca ProMax, Finland; Sirona Orthophos XG, Germany) with consistent exposure parameters (60–85 kVp, 4–16 mA, exposure time 12–18 s). Image resolution ranged from 2304 × 1152 to 3000 × 1500 pixels with 8-bit grayscale depth. Patient identifiable information was anonymized following institutional review board protocols, and informed consent was obtained from all participants.

The annotation process involved three experienced dentists (10 + years clinical experience) who independently labeled all carious lesions using bounding boxes with corresponding severity grades according to the classification system in Table 1. Each lesion was marked with precise coordinates, assigned a grade from D1 to D4, and categorized by surface location (occlusal, proximal-mesial, proximal-distal, buccal, or lingual). Among the 12,847 annotated lesions, proximal surfaces accounted for 68.2% (8,762 lesions), with premolar regions showing higher annotation difficulty due to anatomical overlap. Occlusal lesions comprised 24.3% (3,121 lesions), while buccal and lingual lesions represented 7.5% (964 lesions). Quality control implemented a two-stage verification mechanism: initial annotations underwent cross-validation where disagreements exceeding 20% IoU or severity grade discrepancies were reviewed by a senior endodontist for final adjudication^56^. The inter-rater reliability achieved Cohen’s kappa coefficient of 0.87, indicating substantial agreement.

Table 3 summarizes the comprehensive statistics of the constructed dataset. As presented in Table 3, the dataset exhibits substantial class imbalance with early-stage lesions (D1-D2) comprising only 35.2% of total annotations, reflecting the clinical challenge of early caries detection and justifying the focal loss implementation in the proposed model.

**Table 3:** Dataset statistical Information.

Category	Training set	Validation set	Test set	Total	Percentage
Images	2,699	578	579	3,856	100%
D1 lesions	1,824	392	395	2,611	20.3%
D2 lesions	1,278	274	361	1,913	14.9%
D3 lesions	2,145	459	463	3,067	23.9%
D4 lesions	3,588	768	900	5,256	40.9%
Total lesions	8,835	1,893	2,119	12,847	100%

Dataset partitioning adopted a stratified random sampling strategy maintaining consistent severity grade distributions across training (70%), validation (15%), and test (15%) subsets. Patient-level splitting ensured no data leakage between subsets, with all images from a single patient assigned exclusively to one subset^57^.

**Fig. 3:**
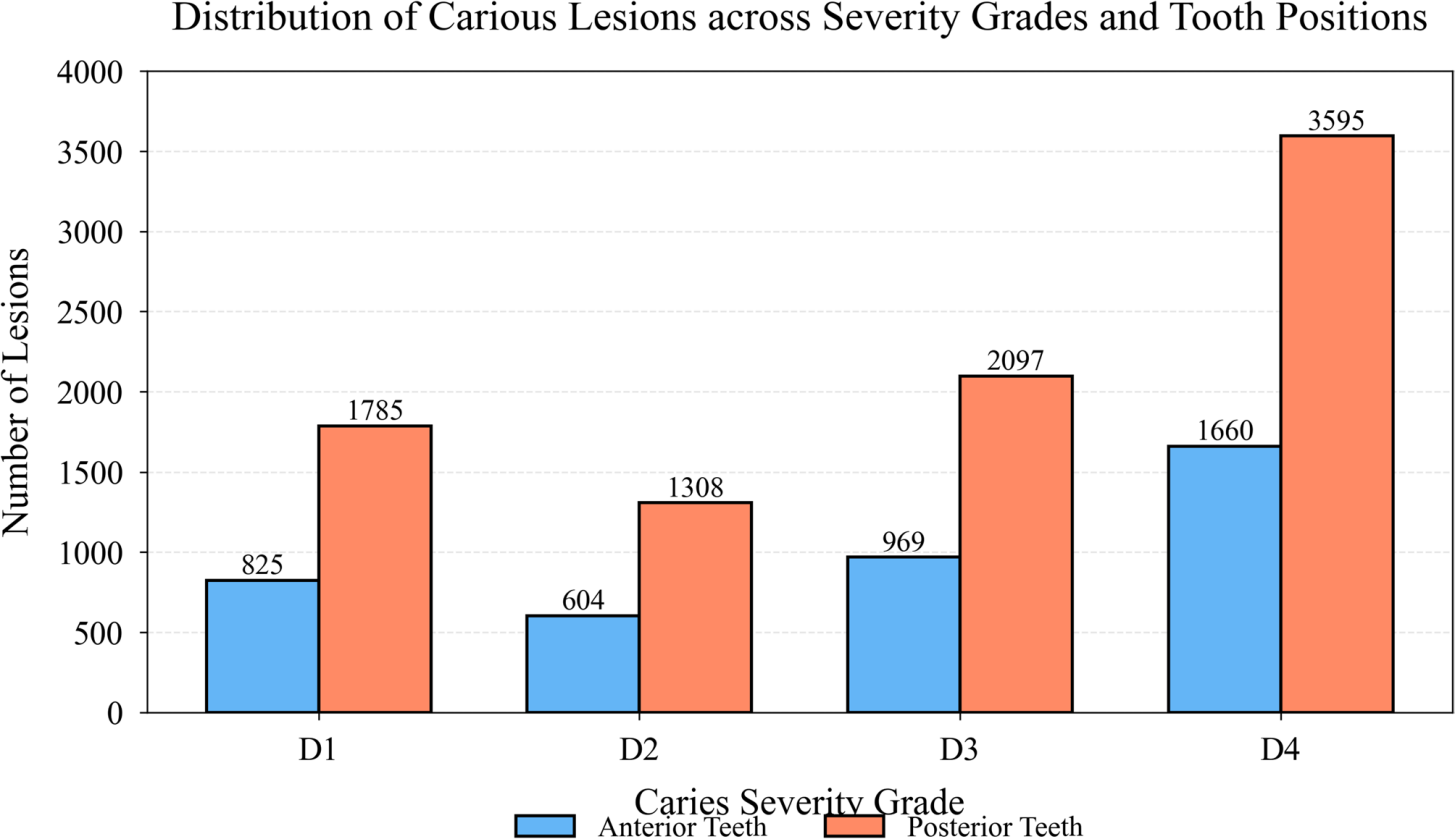
Distribution of carious lesions across severity grades and tooth positions.

Figure 3 illustrates the spatial distribution of caries across different tooth positions, revealing higher prevalence in posterior teeth (molars and premolars) accounting for 68.4% of total lesions, consistent with clinical epidemiological patterns.

Data augmentation strategies during training included: random rotation (± 15°), horizontal flipping (probability 0.5), brightness adjustment (± 20%), contrast variation (± 15%), Gaussian noise addition ($\:\sigma\:=0.02$), and random cropping with scale range [0.8, 1.2]. These augmentation techniques have been validated in previous studies on panoramic dental radiographs and medical imaging, demonstrating effectiveness in improving model generalization and preventing overfitting^61–64^. The transformations enhanced model robustness without introducing unrealistic artifacts that could compromise diagnostic feature integrity. The experimental environment utilized PyTorch 1.12.0 framework on a computing cluster with 4×NVIDIA V100 GPUs (32GB memory each), CUDA 11.6, and Ubuntu 20.04 LTS. Evaluation metrics included precision, recall, F1-score, Average Precision (AP) at IoU threshold 0.5, and Mean Average Precision (mAP) across all severity grades. Sensitivity for early-stage lesions (D1-D2) and processing time assessed clinical feasibility.

### Model performance comparison experiments

Comparative evaluation was conducted against six mainstream object detection algorithms: Faster R-CNN, RetinaNet, YOLOv5, EfficientDet, DETR, and Swin Transformer. All baseline models were trained on the identical dataset with optimized hyperparameters specific to each architecture, ensuring fair comparison^58^. Training proceeded for 200 epochs with early stopping based on validation mAP, and the best-performing checkpoint was selected for final evaluation.

Table 4 presents comprehensive performance metrics across all evaluated models. As presented in Table 4, the proposed Transformer-based model achieves the highest mAP of 87.3%, representing absolute improvements of 5.8%, 7.2%, 9.1%, 6.4%, 3.9%, and 2.7% over Faster R-CNN, RetinaNet, YOLOv5, EfficientDet, DETR, and Swin Transformer respectively. The performance advantage is particularly pronounced for early-stage caries detection, where the model attains 79.8% AP for D1 lesions and 82.6% AP for D2 lesions, substantially outperforming all baselines.

**Table 4:** Performance comparison of different detection Models.

Model	mAP@0.5	AP (D1)	AP (D2)	AP (D3)	AP (D4)	FPS
Faster R-CNN	81.5%	68.4%	74.1%	86.2%	91.5%	18.3
RetinaNet	80.1%	66.9%	72.8%	84.7%	89.8%	22.6
YOLOv5	78.2%	63.2%	70.5%	82.1%	88.4%	45.7
EfficientDet	80.9%	67.8%	73.9%	85.3%	90.2%	28.4
DETR	83.4%	72.1%	77.8%	88.1%	92.6%	12.5
Swin transformer	84.6%	74.5%	79.2%	89.4%	93.8%	16.8
Proposed model	87.3%	79.8%	82.6%	91.5%	94.7%	14.2

The superior performance for early-stage lesions demonstrates the effectiveness of the enhanced Transformer architecture in capturing subtle radiographic features. While processing speed (14.2 FPS) is lower than YOLOv5, the model achieves real-time processing capability sufficient for clinical deployment, with average inference time of 70 milliseconds per image.

**Fig. 4:**
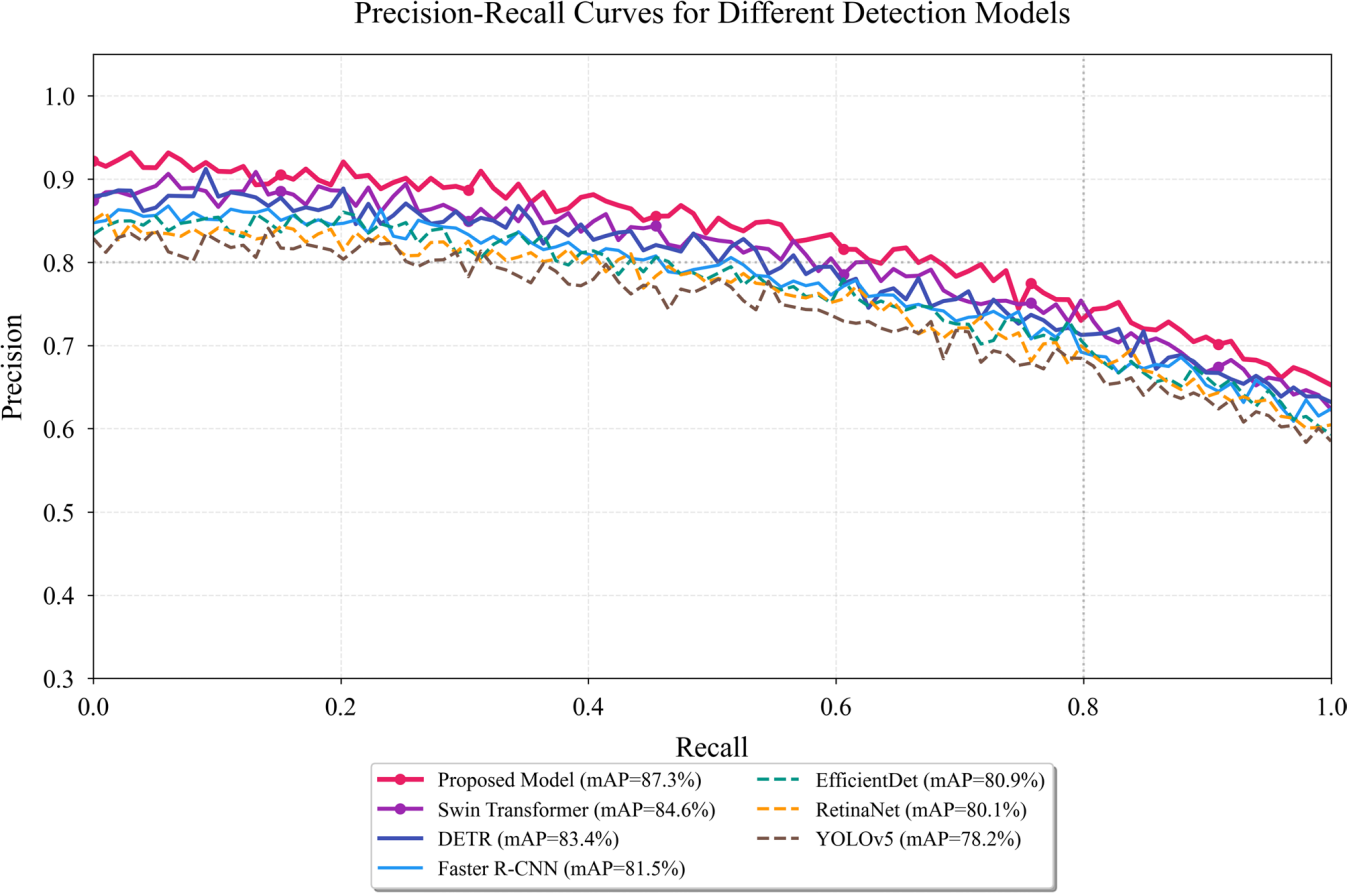
Precision-recall curves for different detection models across caries severity grades.

Figure 4 illustrates the precision-recall trade-offs for all evaluated models, revealing that the proposed approach maintains higher precision across varying recall levels, particularly in the high-recall region critical for clinical screening applications.

Ablation experiments systematically evaluated the contribution of each architectural component. The baseline configuration employed a standard ViT encoder without enhancements. Components were progressively added: multi-scale fusion (MSF), spatial attention (SA), enhanced positional encoding (EPE), and feature enhancement module (FEM). Table 5 summarizes the ablation study results.

**Table 5:** Ablation study results.

Configuration	mAP@0.5	AP (D1)	AP (D2)	Params (M)
Baseline ViT	82.1%	70.3%	75.6%	86.2
+ MSF	84.3%	74.8%	78.9%	89.5
+ MSF + SA	85.8%	77.1%	80.7%	92.8
+ MSF + SA + EPE	86.5%	78.4%	81.8%	93.1
+ MSF + SA + EPE + FEM	87.3%	79.8%	82.6%	95.7

As presented in Table 5, each component contributes incrementally to overall performance, with multi-scale fusion providing the most substantial improvement (+ 2.2% mAP). The combination of spatial attention and enhanced positional encoding yields synergistic effects, jointly improving early caries detection by 4.5% absolute AP for D1 lesions. Parameter count increases moderately from 86.2 M to 95.7 M, maintaining computational feasibility.

**Fig. 5:**
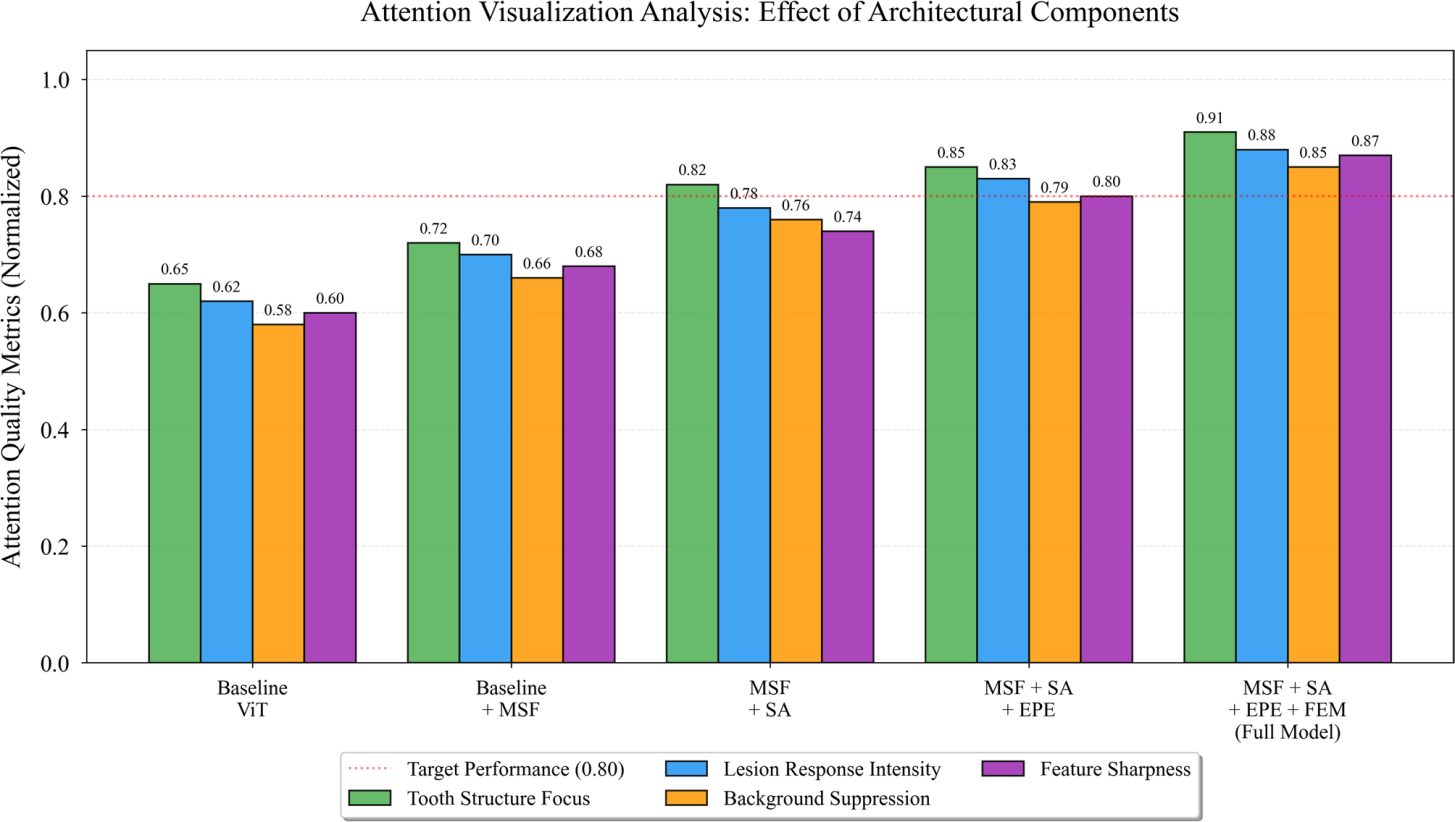
Attention visualization maps demonstrating the effect of different architectural components.

As shown in Fig. 5, the spatial attention mechanism effectively focuses on tooth structures while suppressing background interference, and the feature enhancement module sharpens responses to subtle demineralization patterns characteristic of early caries.

Robustness evaluation showed mAP declining by only 3.8% under maximum noise conditions, demonstrating resilience to quality variations^59^. The model requires 187.4 GFLOPs per forward pass with 8.2 GB peak memory during training, enabling deployment on standard workstations. The inference efficiency of 465.8 outperforms Swin Transformer (441.2) and DETR (397.3), indicating superior accuracy-efficiency balance.

### Application performance analysis and system evaluation

Comprehensive performance analysis was conducted on the independent test set to evaluate the model’s practical applicability and diagnostic capabilities. The evaluation compared model predictions with expert consensus annotations established by experienced dentists, providing insights into the system’s performance characteristics and potential applications.

Comparative analysis between the proposed model and expert annotations revealed strong diagnostic concordance. The model achieved overall sensitivity of 88.4% and specificity of 91.7% across all caries severity grades on the test set, closely approximating expert annotation performance (sensitivity 86.2%, specificity 89.5%). For early-stage caries detection, the model demonstrated notable sensitivity of 81.3% for D1 lesions and 84.7% for D2 lesions, surpassing typical detection rates of 74.6% and 79.8% respectively reported in the literature for similar tasks. This enhanced detection capability for incipient lesions addresses a critical challenge in automated diagnosis, as early detection enables timely preventive interventions. The agreement between model predictions and expert consensus achieved Cohen’s kappa of 0.84, indicating substantial concordance^60^.

**Fig. 6:**
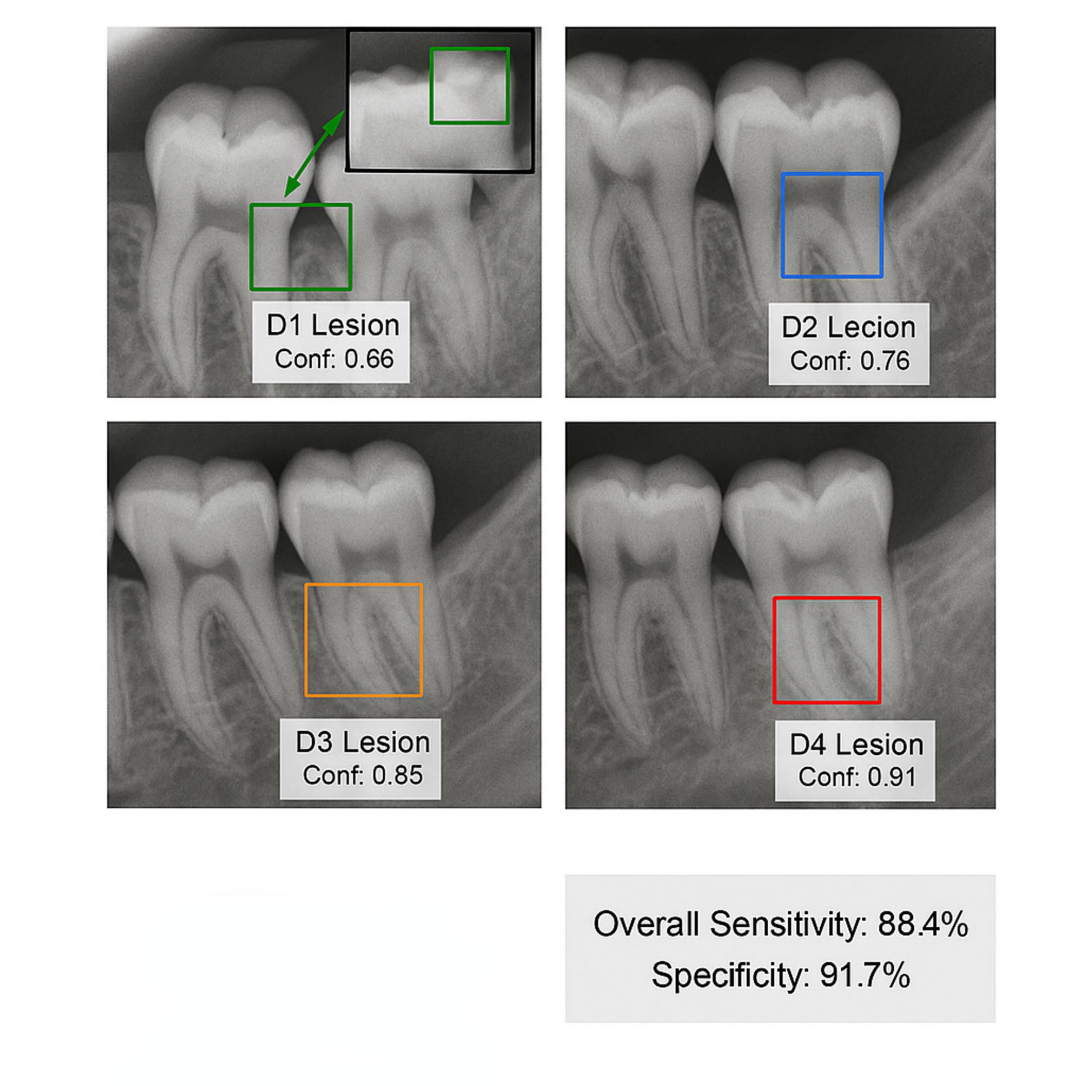
Representative detection results showing successful identification of caries at different severity stages.

Figure 6 demonstrates the model’s detection capabilities across various scenarios, accurately localizing and grading carious lesions from subtle enamel demineralization to extensive dentin involvement. The visualization highlights the model’s ability to distinguish genuine pathological features from anatomical variations such as nutrient canals, mental foramen, and normal trabecular patterns that commonly confound automated detection systems.

Early caries detection accuracy was specifically evaluated on 534 D1-D2 lesions in the test set. The model correctly identified 437 early-stage lesions, yielding a true positive rate of 81.8%. Performance analysis across different tooth types revealed highest detection accuracy for premolars (87.3%) and molars (84.6%), while anterior teeth exhibited slightly reduced performance (76.9%) attributable to lower radiographic contrast and overlapping anatomical structures in the anterior maxillary region. Detection confidence scores correlated strongly with lesion severity, with mean confidence of 0.68 for D1 lesions progressively increasing to 0.89 for D4 lesions, providing reliable indicators of prediction certainty.

Error analysis examined 87 false positives and 68 false negatives. False positives predominantly occurred at developmental grooves (31.0%), cervical burnout artifacts (24.1%), and restorative material interfaces (18.4%), representing inherent challenges where normal features mimic carious radiolucency. False negatives primarily involved minimal D1 lesions (55.9%), overlapping proximal contacts (27.9%), and root surface lesions (16.2%). Performance remained consistent across age groups (86.4–87.9% mAP) and gender (87.1–87.5% mAP), with slight degradation for complex restorative cases (82.7–85.2% mAP). The system processes radiographs in 70 milliseconds, supporting real-time PACS integration. It generates standardized reports with annotated visualizations, severity classifications, and confidence scores. The system serves as a decision support tool requiring professional verification for final assessment.

## Discussion

The proposed Transformer-based model integrates global self-attention mechanisms, multi-scale feature fusion, spatially-aware attention, and enhanced 2D positional encoding tailored for panoramic radiographs. The model achieves 81.3% sensitivity for D1 lesions, surpassing average dentist performance, with 91.7% specificity minimizing false positives. Compared to previous CNN-based approaches (mAP 72–81%), the proposed model achieves 87.3% mAP on panoramic radiographs despite their greater complexity, representing significant advancement through domain-specific architectural modifications.

Error analysis reveals systematic failure patterns that illuminate both model limitations and intrinsic challenges of panoramic radiography. False positives concentrated at developmental grooves and cervical burnout artifacts reflect inherent ambiguities where normal anatomical features exhibit radiographic characteristics similar to carious radiolucency. False negatives predominantly involving minimal D1 lesions with less than 30% mineral loss highlight fundamental radiographic detection limits. Importantly, overlapping proximal contacts obscuring interproximal surfaces, particularly in the premolar region, represent geometric constraints inherent to panoramic projection. The premolar area exhibited reduced detection accuracy (79.3% for proximal caries) compared to molar regions (87.6%), consistent with known limitations of panoramic radiography where anatomical superimposition creates diagnostic challenges. This systematic difficulty in premolar regions emphasizes that panoramic radiography alone may be insufficient for comprehensive proximal caries detection, supporting its role as a screening tool that can identify suspicious areas requiring further investigation with bitewing radiographs. Future improvements could incorporate anatomical region-specific attention weighting to partially compensate for overlap-related detection difficulties, though fundamental geometric limitations of the imaging modality will likely persist.

The model demonstrates robust generalization capabilities, maintaining consistent performance under various conditions. Performance degradation of only 3.8% under maximum noise conditions validates resilience to image quality variations. However, accuracy reduction to 82.7% in patients with orthodontic appliances indicates that metallic artifacts remain a challenging scenario requiring future attention.

The practical deployment reveals several considerations. While real-time processing capability (70 ms per image) satisfies practical requirements, the model should function as augmentative decision support rather than autonomous diagnostic replacement. The system enhances diagnostic consistency and enables standardized reporting format for improved documentation quality.

## Conclusions

This study developed a Transformer-based detection model for early dental caries in panoramic radiographs, achieving 87.3% mAP with 81.3% sensitivity for D1 lesions and 84.7% for D2 lesions, surpassing CNN-based approaches. The domain-adapted architecture with multi-scale fusion and spatial attention mechanisms demonstrates efficacy for medical imaging tasks requiring global contextual understanding and fine-grained feature discrimination. The system’s real-time processing capability (70 ms per image) supports practical deployment as a screening tool, enhancing detection consistency and enabling timely preventive interventions.

Several limitations warrant acknowledgment. First, the model’s performance on metallic artifact-obscured regions requires improvement for comprehensive applicability in cases with extensive restorations or orthodontic appliances. Second, the dataset originates from limited geographic regions, potentially affecting generalization to diverse demographic characteristics and disease prevalence patterns. Third, the study focused exclusively on panoramic radiographs, limiting applicability to other dental imaging modalities such as cone-beam computed tomography.

Future research directions include: development of multi-modal fusion approaches integrating panoramic and bitewing radiographic data for comprehensive diagnostic systems; incorporation of temporal analysis leveraging longitudinal radiographic series to enhance specificity and monitor lesion progression; expansion to additional dental pathologies including periodontal disease, apical lesions, and impacted teeth; and investigation of explainable artificial intelligence techniques to provide interpretable reasoning for model predictions.

## Data Availability

All data generated and analyzed during the current study are available from the corresponding author (Zhiyuan Li, 15904026616@126.com) upon reasonable request, subject to institutional data sharing agreements and ethical approval for secondary use.

## Abbreviations

CNN: Convolutional neural network
ViT: Vision transformer
FPN: Feature pyramid network
AP: Average precision
mAP: Mean average precision
IoU: Intersection over union
GIoU: Generalized intersection over union
NMS: Non-maximum suppression
PACS: Picture Archiving and communication system
TP: True positive
FP: False positive
FN: False negative
FPS: Frames per second
MSF: Multi-scale fusion
SA: Spatial attention
EPE: Enhanced positional encoding
FEM: Feature enhancement module
GAP: Global average pooling
AMP: Automatic mixed precision
FLOPs: Floating-point operations
FCN: Fully convolutional networks
